# Attitudes of Critical Care Nurses towards Teamwork and Patient Safety in Saudi Arabia: A Descriptive Cross-Sectional Assessment

**DOI:** 10.3390/healthcare10101866

**Published:** 2022-09-25

**Authors:** Fatchima L. Moussa, Mahaman Moussa, Hussain Ahmed Sofyani, Bander Hammad Alblowi, Yahia Ahmad Oqdi, Saleh Khallaf, Hamad S. Alharbi, Ahmed Albarqi

**Affiliations:** 1 Medical Surgical Department, College of Nursing, Princess Nourah Bint Abdulrahman University, Riyadh 11564, Saudi Arabia; 2 College of Nursing, King Saud University, Riyadh 11451, Saudi Arabia; 3 Ohud General Hospital, Ministry of Health, Medina 42354, Saudi Arabia; 4 Almeqat General Hospital, Ministry of Health, Medina 42373, Saudi Arabia; 5 Alansar Hospital, Ministry of Health, Medina 42644, Saudi Arabia; 6 Hanakyah General Hospital, Ministry of Health, Jeddah 23436, Saudi Arabia

**Keywords:** teamwork, safety attitudes, critical care nurses, Saudi Arabia

## Abstract

The study aimed to assess the teamwork and safety attitudes among the critical care unit (CCU) nurses in Saudi Arabia. A descriptive cross-sectional study was carried out in public tertiary hospitals in Al-Madinah, Saudi Arabia. All participants answered a three-part questionnaire that included demographic data, a teamwork attitude questionnaire (T-TAQ), and the Safety Attitudes Questionnaire (SAQ). The analysis revealed that the majority of the nurses were female, *n* = 52 (76.5%), and almost half of the nurses were aged from 29 to 39 years, *n* = 29 (42.6%). Teamwork attitude values are found to be relatively stable in all subscales, ranging from 1.63 (SD = 1.23) to higher at 2.92 (SD = 1.32). Of the six dimensions of SAQ, job satisfaction (M = 70, SD 21.46) had the highest positive rate and was approached with a positive attitude, followed by teamwork (M = 66.09, SD 15.12) and safety climate (M = 67.11, SD 17.70). The analysis also shows work experience was the influencing factor of teamwork attitude and safety attitude of nurses, recording beta values of 0.24, *p* < 0.05 and 0.10, *p* < 0.001, respectively. The results also identified an association between teamwork and safety attitudes. The study reflected the positive attitudes towards teamwork and less positive attitudes toward patient safety among critical care nurses in Saudi Arabia. Collaborative team performance among nurses improves the medical care quality and patients’ safety, decreasing the occurrence rate of adverse events.

## 1. Introduction

Critical care nurses are highly specialized healthcare providers who are exposed to very stressful situations and face challenging responsibilities that demand fulfillment of the highest quality of healthcare [1]. Working with the most severely ill hospitalized patients can be overwhelming for nurses because of the complexity and needs of multitasking and heavy workloads [2]. Furthermore, the nursing workforce in Saudi Arabia is composed of different nationalities. According to the Ministry of Health’s records, 66% of the total population of nurses were expatriates, such as Indians, Filipinos, and Malaysians [3]. Since the largest percentage of the nursing workforce in Saudi Arabia is provided by expatriates, different cultural beliefs, attitudes, and behaviors are prevalent [4,5]. Differences can also occur in the patients and other Saudi colleagues, which might hinder nurse–patient and nurse–employer interaction [4]. Working in a multi-cultural environment also limits an employee’s potential because of barriers such as communication, different work cultures, decision-making conflicts, and unconscious cultural biases [4,6]. Dealing with all these barriers requires significant effort and collaboration between nurses. If these differences are not handled effectively, they could affect the quality of care and patient safety.

Several studies reported that teamwork among healthcare providers improves the quality of care and patient safety by creating an environment that works together with a common goal [7,8,9]. The concept of patient safety in healthcare is well established [10,11]. In addition, team performance is crucial in providing safe patient care [7,12,13]. Delays in treatment and poor coordination among providers can affect the quality of care and patient safety [14]. Consequently, the occurrence of medical errors can be a risk, which can lead to patient harm. A report showed a 37% increase in medical error claims in Saudi Arabia over five years from 2011 to 2016 [15]. Effective teamwork reduces medical errors and improves the quality of care and patient safety [16]. A collaborative interprofessional team composed of physicians, nurses, and other allied health professionals has been embraced in critical care medicine to provide a system-based intervention for quality care and improve patient safety [16].

Teamwork is vital to healthcare delivery. There are several units in nursing service, such as the intensive care unit, operating room, emergency, and trauma unit. These units work under extreme pressure with frequently changing and rotating team members, having a short period of working time, and requiring several professionals in a unit. The attitudes of each professional within these units may become more challenging because of workload and working conditions [17]. Moreover, effective teamwork must be achieved to ensure a patient-centered and quality healthcare service [7].

Nurses constitute a large part of a healthcare team because they care for patients directly. Thus, it is important to determine nurses’ attitudes toward teamwork and safety factors. When investigating effective teamwork, it is important to know the perceptions of teamwork among different members. This study aims to assess the teamwork and safety attitudes among the critical care unit (CCU) nurses in Saudi Arabia.

## 2. Materials and Methods

### 2.1. Design and Setting

A descriptive cross-sectional study was carried out in public tertiary hospitals in Al-Madinah, Saudi Arabia. The Institutional Review Board of the General Directorate of Health Affairs in Madinah approved the study (IRB no: 222-143-3-1440).

### 2.2. Participants

All nurses working in the critical care unit were invited to participate in this study. Nurses who worked outside the critical care units or did not accept the study were excluded from the study sample. The sample size required for this study was calculated by using the G-power program, with a power of 80%, where α was set at 0.05 and a small effect size of 0.10. The minimum sample size was calculated to be 80, with an additional 25% to compensate for nonresponse.

### 2.3. Procedure

After we received the approval of the study protocol from the General Directorate of Health Affairs in Madinah, we sought the permission of the hospital administrators and nursing directors at the hospitals. The principal investigator met with the unit managers and explained the objectives of the study. Two investigators facilitated the distribution of the questionnaire. The investigators explained the study’s aims, and they had the right to decide whether to answer or complete the survey. Study protocols such as confidential and anonymous handling of the data and their right to withdraw at any point in the research were also communicated to the participants before distributing the questionnaire. A sealed questionnaire with consent was also given to the unit managers for critical care nurses who were off duty. After three weeks, envelopes were collected by the investigators.

### 2.4. Measures

The first part included questions on sociodemographic characteristics such as gender, age, educational status, marital status, and work experience. The second part of the questionnaire was the Teamwork Attitude Questionnaire (T-TAQ) from TeamSTEPPS, which was used to assess the attitude of nurses in the critical care unit towards teamwork [18]. The scale consists of 30 items that assess five dimensions of teamwork, with six questions in each dimension. The five dimensions include team structure, leadership, situation monitoring, mutual support, and communication. The items were scored using a 5-point Likert scale from strongly disagree (1) to strongly agree (5). The scale has an acceptable internal consistency with a Cronbach’s alpha of 0.773 and a CFI of 0.794 [18,19].

The third part of the questionnaire was the Safety Attitudes Questionnaire (SAQ) developed by Sexton (2006) [20]. The SAQ comprises of 36 items that measure six variables (teamwork climate, safety climate, job satisfaction, stress recognition, unit management, and work conditions). The SAQ was used in previous studies that assessed the safety culture and yielded a strong psychometric property [18,19,20]. The SAQ scores were transformed using the following equation: (mean dimension score − 1) × 25 = the mean score. A score of 75 and above indicates a positive attitude consistent with previous research [20]. All participants answered the questionnaire using a 5-point Likert scale ranging from strongly disagree (1) to strongly agree (5).

### 2.5. Statistical Analysis

Statistical analysis was performed with SPSS, version 23.0 (Chicago, IL, USA). Data were represented using descriptive statistics, and categorical and other quantifiable variables were described using frequency and percentage tables, means, and standard deviations (SD). The correlation between teamwork attitude and safety attitude was analyzed using Spearman’s correlation. Multiple regression analysis was used to analyze the factors associated with teamwork and safety attitude. The statistical significance was determined at a *p*-value < 0.05 level.

## 3. Results

Of the 100 questionnaires distributed to the participants, 68 (107 males and 91 females) were completed and retrieved (a 68% response rate). Table 1 presents the demographic characteristics of the sample. The analysis revealed that the majority of the nurses were female, *n* = 52 (76.5%), and almost half of the nurses were aged from 29 to 39 years, *n* = 29 (42.6%). The educational status of the nurses revealed that more than half of them, *n* = 37 (54.4%), had a bachelor’s degree and were single, *n* = 42 (61.8%). The average nursing experience revealed that most had almost 5 to 10 years of experience, *n* = 36 (53%). Table 2 presents the item analysis of the T-TAQ scale. Of all dimensions of the teamwork attitude scale, simulation monitoring and mutual support had the highest and lowest scores, respectively (25.61 and 18.12). The mean score of team attitude in all five dimensions was as follows: team structure (24.50), leadership (23.75), simulation monitoring (25.61), mutual support (18.12), communication (19.25), and overall (114.23).

The scores in the six dimensions of the SAQ are presented in Table 3. Of the six dimensions of SAQ, job satisfaction (M = 70, SD 21.46) had the highest positive rate and was approached with a positive attitude, followed by teamwork (M = 66.09, SD 15.12) and safety climate (M = 67.11, SD 17.70). Meanwhile, the lowest number of negative responses were stress recognition (M = 57.01, SD 19.2) and hospital/unit management (M = 62.43, SD 16.57). Table 4 presents the correlation between teamwork and safety attitudes in critical care units. It depicts a significant relationship between teamwork attitude and safety attitude observed in critical care units (*p* ≤ 0.001).

Table 5 shows the factors associated with the teamwork of nurses. The multiple regression analysis showed that work experience was the influencing factor of teamwork attitude and safety attitude of nurses, recording beta values of 0.24, *p* < 0.05 and 0.10, *p* < 0.001, respectively. In addition, gender was statistically significant with the safety attitude of nurses, accounting for a beta value of 0.11, *p* < 0.05.

## 4. Discussion

The study provides insight into the attitudes of critical care unit nurses toward teamwork and patient safety in Saudi Arabia. The findings revealed a moderate to high level of attitudes towards teamwork that indicates a positive attitude among critical care nurses in Saudi Arabia. The results are similar to previous studies of clinical teams in Iran and nurses and surgeons in Scotland [21,22]. Several studies revealed that teamwork among nurses improves the patient’s health outcomes, team communication, responsiveness to adverse events, healthcare staff and patient satisfaction, and decreases medical errors [13,23,24,25,26]. For example, Epstein (2014) et al. showed that cohesive teamwork improves team communication, its responsiveness to adverse events, and yields greater staff satisfaction [24]. Furthermore, Burgerer (2020) et al. suggest that effective communication among healthcare workers increases patient satisfaction and decreases errors in medical care [25]. The positive attitudes displayed by the nurses indicate their awareness, collaboration, and adaptability among the team members. Failure to build effective teamwork could lead to unnecessary clinical issues and errors with detrimental consequences [27,28]. It is essential to assess healthcare providers’ attitudes, particularly in a critical care unit with multiple teams. Healthcare providers in critical care units require a high level of coordination and interprofessional communication. Enhancing teamwork attitudes among healthcare providers is necessary to provide and ensure quality patient care [27].

The present study revealed nurses’ highest mean score attitudes towards teamwork in the dimension of situational monitoring and communication. The use of situational care has been shown to be an effective tool for responding to critical situations [28]. Figueroa et al. (2013) support the study results and highlight that confidence and communication in a multidisciplinary team improve when situational monitoring is practiced. Situation monitoring is different from watching teammates doing their work [29]. Situation monitoring is described as an awareness of the interconnected team performance and functioning and its environment [30]. Members of the healthcare team, particularly in the critical care unit, need to see the whole situation and how their other members perform to make necessary adjustments and form an effective healthcare team.

Moreover, effective communication is essential in creating safe and quality care. Active communication may reduce conflict, lessen stress among team members, and promote healthy communication [31,32]. Different strategies, such as teamwork promotion, are necessary to ensure nurses’ competency in different circumstances. For example, information-dependent approaches such as interactive workshops, simulation exercises, and role-playing are strategies that can be helpful tools to improve teamwork among healthcare providers [23,33].

Concerning safety attitudes, the study revealed that nurses have less than positive attitudes toward patient safety. The current findings are parallel with previous research in Saudi Arabia among nurses and doctors, particularly in the domains of stress recognition and perceptions of management [34]. The findings are consistent with studies conducted in Sweden and Brazil among healthcare providers [35,36]. Although the percentage was higher than in the previous studies, negative attitudes may cause and create vulnerabilities to medical errors. The negative attitude may be due to factors such as quality of work environment, work conditions (e.g., workload), hospital management, and job satisfaction.

The present study also highlights the significant association between teamwork and nurses’ safety attitudes. Although the limited studies could establish a direct association between teamwork and safety attitudes, the present finding could be added to the training of nurses to improve the team and develop effective teamwork and safe patient care. Interestingly, the predictors are associated with nurses’ attitudes toward teamwork and safety. Identifying the association may help policymakers and administrators develop strategies and interventions to enhance nurse teamwork. The results showed that work experience was significantly associated with nurses’ attitudes towards teamwork and safety. This indicates the work experience of nurses was a predictor in the effectiveness among the team members and may assist in overcoming the barriers which affect the delivery of quality care to the patient. It depicts that teamwork is impacted as a result of team attitude.

The study presents some limitations. First, the study’s small sample size limits the generalizability of the results. Second, the study was conducted in a specific region and used a convenience sampling technique which may have led to the underrepresentation of critical care nurses in Saudi Arabia. Lastly, the study used a cross-sectional design, which inferred causality. Nevertheless, the study used a validated questionnaire, and these new findings regarding attitudes towards teamwork and patient safety can substantially contribute to the limited literature on this topic.

## 5. Conclusions

In conclusion, the study provides information about nurses’ attitudes toward teamwork and safety attitudes. This study reflected the positive attitudes towards teamwork and less positive attitudes toward patient safety among critical care nurses in Saudi Arabia. Notably, the study identifies domains or services that can improve according to the attitudes among critical care nurses. The study revealed a negative perception of all safety domains, identifying areas requiring enhancement and development. The results also identified an association between teamwork and safety attitudes. The work experience of nurses revealed a significant predictor of teamwork and safety attitudes, and this shows that nurses’ work experience impacts the effectiveness of teamwork and safety attitudes. Finally, the study suggests further research is needed to focus on more precisely identifying the relationship between teamwork attitudes and hospital error rates. Collaborative team performance among nurses improves the medical care quality, patients’ safety, and decreases the occurrence rate of adverse events. The hospital needs to implement training initiatives where a simulated learning environment facilitates better learning without causing any potential harm to the patient.

## Figures and Tables

**Table 1:** Participants demographic characteristics.

Variables		Frequency (*n*)	Percentage (%)
gender			
	female	52	76.5
	male	16	23.5
age			
	18 to 28 years	28	41.2
	29 to 39 years	29	42.6
	40 years and above	11	16.2
education			
	graduate	37	54.4
	master’s	19	27.9
	diploma	12	17.6
marital status			
	single	42	61.8
	married	13	19.1
	others	13	19.1
work experience			
	1 to 4 years	22	32.3
	5 to 10 years	36	53.0
	10 years and above	10	14.7

**Table 2:** TAQ subscale mean scores of nurses.

TAQ and Its Subscales	TAQ Score Range Min–Max	TAQ Scores Mean
team structure	6–30	24.50
leadership	6–30	23.75
simulation monitoring	6–30	25.61
mutual support	5–25	18.12
communication	5–25	19.25
TAQ total score	28–140	114.23

**Table 3:** Attitude of nurses regarding patient safety (SAQ).

SAQ Subscales	Mean	SD
teamwork climate	66.09	15.12
safety climate	67.11	17.70
job satisfaction	70.49	21.46
stress recognition	57.01	19.26
hospital/unit management	62.43	16.57
working conditions	63.14	20.50

**Table 4:** Correlation among the factors affecting teamwork attitude and safety attitude.

		Safety Attitude
teamwork attitude	Correlation Coefficient	0.451 **
	Sig. (2-tailed)	0.0001
	*N*	68

** Correlation is significant at the 0.01 level (2-tailed).

**Table 5:** Multiple regression analysis of the factors affecting teamwork attitude and safety attitude of nurses.

	Teamwork Attitude			Safety Attitude		
Variables	B	95% CI	*p* Value	B	95% CI	*p* Value
Gender	0.46	0.12–1.0	0.076	0.11	0.01–0.24	**0.021**
Age	−0.53	−0.51–−0.29	0.544	0.17	0.01–0.32	0.829
Education	0.21	0.02–1.12	**0.041**	0.14	0.04–0.26	0.568
Marital Status	−0.62	−0.58–−0.43	0.053	0.26	0.08–0.42	0.624
Work Experience	0.24	0.01–1.20	**0.018**	0.10	0.06–0.15	**0.001**

Note: Bold numbers are considered significant. Significant difference level *p* < 0.05.

## Data Availability

The data set used is locked and stored at College of Nursing, Medical Surgical Department, Princess Nourah Bint Abdulrahaman University and can be obtained from the author on reasonable request.
